# A Good Thing for Boys

**Published:** 1889-07

**Authors:** 


### A GOOD THING FOR BOYS.

Manual training is one of the few good things that are good for everybody. It is good for the rich boy, to teach him respect for the dignity of beautiful work. It is good for the poor boy, to increase his facility for handling tools, if tools prove to be the things, he must handle for a living, afterward. It is good for the bookish boy, to draw him away from his books. But, most of all, it is good for the non-bookish boy; it shows him that there is something he can do well. The boy utterly unable, even if he were studious, to keep up in book knowledge and percentage with the brighter boys, becomes discouraged, dull, and moody. Let him go to the work-room for an hour, and find that he can make a box or plane a rough piece of board as well as the brighter scholar, yes, very likely better than his brighter neighbor, and you have given him an impulse of self-respect that is of untold benefit to him when he goes back to his studies. He will be a brighter and better boy for finding out something that he can do well. Mind you, it is planing the board in the presence of other boys who can no longer look down upon him when they see how well he can plane. He might go home after school, and plane a board in the bosom of his family, or go to an evening school to learn to plane, without a quarter part, nay, without any of the invaluable effects upon his manhood that it will have to let him plane side by side with those who in mental attainments may be his superiors. *American Magazine.*

